# Two-Stage Microporous Layers with Gradient Pore Size Structure for Improving the Performance of Proton Exchange Membrane Fuel Cells

**DOI:** 10.3390/polym15122740

**Published:** 2023-06-19

**Authors:** Chongxue Zhao, Haihang Zhang, Zheng Huang, Meng Zhao, Haiming Chen, Guangyi Lin

**Affiliations:** College of Mechanical and Electrical Engineering, Qingdao University of Science and Technology, Qingdao 266061, China

**Keywords:** proton exchange membrane fuel cell, gradient gas diffusion layer, pore structure, water management, gas transmission

## Abstract

In this paper, we report the preparation of a gas diffusion layer (GDL) with different gradient pore size structures. The pore structure of microporous layers (MPL) was controlled by the amount of pore-making agent sodium bicarbonate (NaHCO_3_). We investigated the effects of the two-stage MPL and the different pore size structures in the two-stage MPL on the performance of proton exchange membrane fuel cells (PEMFC). The conductivity and water contact angle tests showed that the GDL had outstanding conductivity and good hydrophobicity. The results of the pore size distribution test indicated that introducing a pore-making agent altered the pore size distribution of the GDL and increased the capillary pressure difference within the GDL. Specifically, there was an increase in pore size within the 7–20 μm and 20–50 μm ranges, which improved the stability of water and gas transmission within the fuel cell. The maximum power density of the GDL03 was increased by 37.1% at 40% humidity, 38.9% at 60% humidity, and 36.5% at 100% humidity when compared to the commercial GDL29BC in a hydrogen-air environment. The design of gradient MPL ensured that the pore size between carbon paper and MPL changed from an initially abrupt state to a smooth transition state, which significantly improved the water and gas management capabilities of PEMFC.

## 1. Introduction

The proton exchange membrane fuel cell (PEMFC) has received attention worldwide because of its non-pollution, low operating temperature, high conversion efficiency, and durability. It has been recognized as an emerging energy source for stationary and mobile applications and transportation [1,2,3,4]. The single-cell structure of PEMFC is mainly composed of an anode and cathode bipolar plate (BPP) and membrane electrode assembly (MEA); MEA is formed of anode and cathode gas diffusion layer (GDL), anode and cathode catalyst layer (CL) and proton exchange membrane (PEM) [5,6]. The PEMFC works as follows: H_2_ and O_2_ reach the anode and cathode CL via the anode and cathode BPP and GDL, respectively. To achieve proton transport, H_2_ is oxidized to H^+^ and e^−^ (H_2_ → 2H^+^ + 2e^−^), e^−^ arrives at the cathode via the external circuit, and H^+^ travels to the cathode via the PEM in the form of hydronium ions (H_3_O^+^). H_3_O^+^ and e^−^ combined with O_2_ on the CL side of the cathode to form water (O_2_ + 4H^+^ + 4e^−^ → 2H_2_O) and release energy [7]. GDL is used as a carbon-based material between the flow field plate and CL on both sides of the fuel cell anode and cathode, and it usually consists of a microporous layer (MPL) and a base layer (GDBL) made of porous carbon paper or carbon cloth. The GDL’s main functions include: facilitating the movement and distribution of the reactant gases H_2_ and O_2,_ as well as the product water; supporting the membrane electrode mechanically; and transferring electrons and heat [8,9]. The cathode CL will produce large amounts of water at high current densities as the gas reaction accelerates. This water must be transported over time through the pore size of GDL and finally expelled from the fuel cell through the cathode flow channel. To avoid flooding or reaction, gas cannot be transported to CL; it is necessary to design and prepare GDL with a good balance of water and gas management [10].

The structure and characteristics of MPL significantly influence the output performance of the fuel cell. MPL has been found to improve fuel cell performance at high and moderate current densities [11,12]. The performance of MPL is affected by many factors, including the type and loading process of toners, type, and content of hydrophilic and hydrophobic agents, pore structure and thickness of MPL, etc. The above factors are used to optimize MPL and improve GDL’s water and gas management capabilities. Some researchers altered the porosity and pore size distributions in MPL to improve PEMFC performance [13,14]. Chun et al. [15] used thermal-expanded graphite as MPL material to change the pore structure of GDL; it only analyzed the relationship between current and voltage at 100% high humidity, and lacked the analysis of current density and power density at low and medium humidity conditions. In addition, there was a lack of comparative experimental groups for gradient MPL and no further explanation of the capillary pressure mechanism within the MPL. In this study, more comprehensive and rational experiments and analyses were conducted. It has been demonstrated that increasing the hydrophilic carbon black content in MPL positively impacted fuel cell power density [16,17]. Other researchers studied the water transport properties of pores with various sizes and spacing using laser perforations of GDL [18]. Ren et al. [19] prepared GDL with nanoscale gradient pore size through electrospinning to explore the influence of gradient layers and the degree of pore size gradient on water transport performance. It used electrostatic spinning technology to prepare GDL with nanoscale gradient pore size. The electrostatic spinning process was costly and complicated to operate, and the spraying method, which was a simple and inexpensive preparation process, was used in this experiment. Some researchers found that porosity gradients in MPLs also enhanced water removal properties, thereby improving fuel cell performance. Turkmen et al. [20] used GDL with high porosity to improve the output performance of fuel cells and modeled and validated all gas diffusion plates with fixed properties but different porosity. Some researchers improved the water management status of cathodes by changing the hydrophilic and hydrophobic properties of MPL. Morgan et al. [21] investigated the effects of adding multiple MPLs, MPL loading, and MPL particle size on fuel cell performance under wet and dry conditions. To address durability issues caused by carbon corrosion in GDL of PEMFC porous carbon paper, researchers prepared a Cr_7_C_3_ ceramic coating on absorbent carbon paper by the molten salt method [22]. Some researchers grew Pt nanowires (Pt-NWs) in situ to prepare double-layer MPLs [23]. Several researchers worked on anode GDLs and found that modified GDLs containing a hydrophilic TiO_2_ layer between MPL and GDBL exhibited better self-humidification properties than conventional GDLs without a TiO_2_ layer [24]. Brahim Laoun et al. [25] increased the hydrophobicity of GDL by increasing the amount of PTFE to improve the performance of PEMFC. Latorrata et al. [26] found that fluorinated ethylene propylene (FEP), perfluoroalkoxy (PFA), and perfluoropolyether (PFPE) were superior to polytetrafluoroethylene (PTFE) in the improvement of fuel cell performance. Some researchers improved drainage was favored by increasing the PTFE concentration in the GDL inlet region [27]. The thickness of the GDL was also one of the critical factors affecting the water and gas management of the GDL. Experimental studies on the effect of GDL thickness showed that an optimal MPL thickness existed to achieve optimal fuel cell performance [28]. Bahar Amani et al. [29] improved the performance of the baffle channel by blocking the cathode channel to increase the thickness and zigzag degree of GDL while reducing its porosity and permeability. Lin et al. [30] designed a double-layer MPL structure GDL and studied the effect of double MPL thickness on PEMFC performance. They used two different conductive materials to prepare the GDL to investigate the effect on the water and gas management of the PEMFC. However, the electrochemical tests of cathodic were performed under 100% O_2_ and there was a lack of electrochemical tests and analysis under low humidity conditions. This work was tested at three different humidity levels, low, medium and high, and the electrochemical tests of cathodic were performed in an air environment, which was more convincing and practical guidance.

In this work, two layers of MPL with different pore-size structures were prepared based on carbon paper by adding the pore-making agent NaHCO_3_. The porosity of each layer was controlled by the amount of a pore-making agent so that the pore diameter gradually increased from the CL/MPL interface to GDBL. The porosity gradient was designed primarily to increase the driving force exerted on the droplet. Eventually, a good balance between drainage and gas transport was established.

## 2. Experimental

### 2.1. Materials

Carbon paper (Toray Group, Tokyo, Japan; TGPH-060), anhydrous ethanol (Sinopharm Chemical Reagent Co., Ltd., Shanghai, China; purity ≥ 99.7%), carbon black (Vulcan XC-72, Cabot Corporation, Boston, MA, USA), PTFE (60 wt.%, Denka, Tokyo, Japan), GDL29BC (thickness 235 μm, Sigracet Group, Wiesbaden, Germany), catalyst coated membrane (CCM, active area 2 × 2 cm, Pt loading: anode 0.1 mg/cm^2^, cathode 0.2 mg/cm^2^, Wuhan Ximalaya Co., Ltd., Wuhan, China), sodium bicarbonate (NaHCO_3_, Sinopharm Chemical Reagent Co., Ltd., Shanghai, China). All the materials were used without further treatment.

### 2.2. Assemblage of Two-Stage Gradient MPL

**Figure 1:**
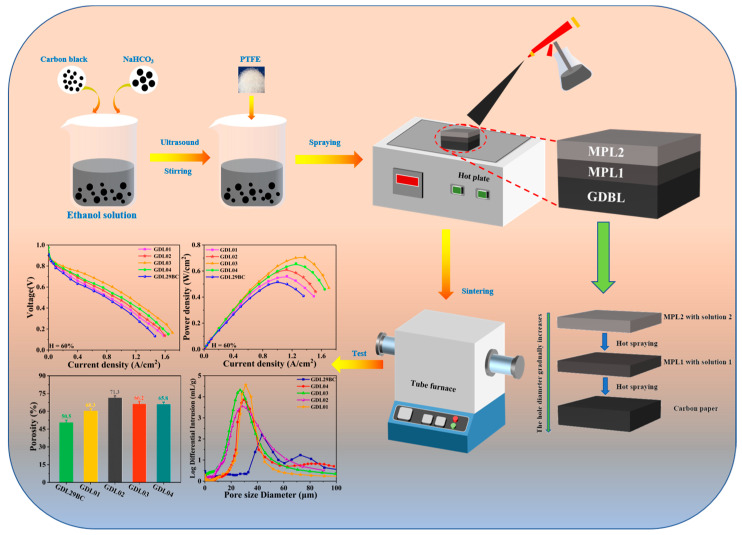
Process of GDL sample preparation.

The preparation of GDL consisted mainly of the preparation and mixing of the solution, coating of MPL, and sintering of GDL. A diagram of the preparation of GDL is shown in Figure 1. First, Vulcan XC-72 conductive carbon black and NaHCO_3_ powder were added to the anhydrous ethanol. After mixing evenly, the PTFE emulsion was added. After several ultrasonic dispersions and magnetic stirring, mixed solutions of carbon black with different mass fractions of NaHCO_3_ were obtained. Two-stage MPLs with different pore sizes and distributions were prepared by spraying the mixed solution on carbon paper and then sintering it at high temperatures. The following is the specific preparation process for the two-stage MPL.

In the first step, a certain amount of Vulcan XC-72 conductive carbon black and NaHCO_3_ powder was added to the anhydrous ethanol and stirred on a magnetic stirrer for 30 min, followed by ultrasonic treatment for 30 min. This process was repeated five times. The aim was to disperse Vulcan XC-72 conductive carbon black and NaHCO_3_ powder uniformly in anhydrous ethanol. The PTFE emulsion was added to the above solution, which was ultrasonic for 30 min and stirred for 30 min. The process was repeated twice. The prepared solution was named solution No. 1, where NaHCO_3_ to the total mass of Vulcan XC-72 and PTFE was 1:2, and PTFE mass fraction was 30%. Prepared another solution according to the above preparation process, where NaHCO_3_ to the total mass of Vulcan XC-72 and PTFE was 1:4. The resulting solution was named solution No. 2, where the contents of Vulcan XC-72 and PTFE emulsions were the same as solution No. 1. The second step was to spray an anhydrous ethanol solution of NaHCO_3_ powder on the carbon paper with a spray gun. The spraying process was carried out on an electric heating plate to evaporate excess anhydrous ethanol. The thickness of the spraying was 30 μm, and consistency was measured by a digital thickness meter with an accuracy of 1 μm. On this basis, another solution of NaHCO_3_ powder with different mass fractions was sprayed with a coating thickness of 30 μm. At the same time, a layer of MPL with a thickness of 60 μm was sprayed on another carbon paper. In the final step, the sprayed GDL was placed in a tubular furnace for sintering. The specific sintering process was from room temperature at a heating rate of 5 °C/min to 250 °C held for 30 min to remove the surfactant of the PTFE. Then, heated to 350 °C at 2 °C/min for 30 min to melt PTFE and finally reduced to room temperature at 5 °C/min. After completing the above three steps, a gradient microporous layer with hydrophobic and conductive properties was prepared. The names and detailed parameters of the different GDL samples are given in Table 1.

**Figure 2:**
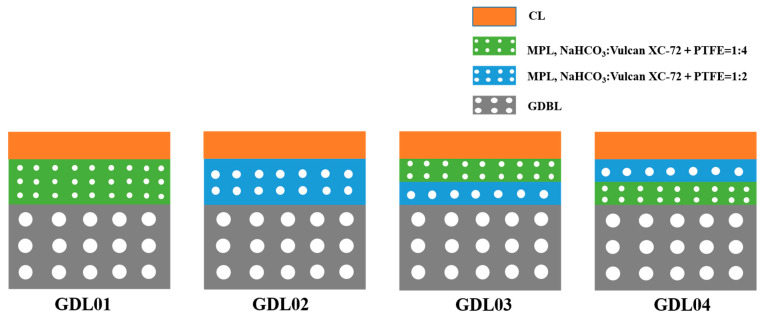
Schematic diagram of the structure of GDL with different gradients.

In this experiment, four kinds of GDL with gradient pore size structures were prepared. The MPL with a thickness of 60 μm sprayed with solution No. 2 on carbon paper was defined as GDL01. MPL with a thickness of 60 μm sprayed on carbon paper with solution No. 1 was defined as GDL02. MPL1 with a thickness of 30 μm was first poured on carbon paper using solution No. 1, and MPL2 with a thickness of 30 μm was spread on top of MPL1 using solution No. 2, and the resulting GDL was defined as GDL03. MPL1 with a thickness of 30 μm was first spread on carbon paper using solution No. 2, and MPL2 with a thickness of 30 μm was sprinkled on top of MPL1 using solution No. 1, and the resulting GDL was defined as GDL04. Its specific structure is shown in Figure 2.

### 2.3. Characterization and Test Methods

The surface morphology and section morphology of GDL were obtained by scanning electron microscope (SEM; Hitachi S-4700, Tokyo, Japan). A tubular furnace (TL 1200; Nanjing Boyuntong Instrument Technology Co., Ltd., Nanjing, China) was used to obtain the sintered GDL. The water contact angle instrument (TBU 90E; Data Physical Instruments Deutschland, Stuttgart, Germany) tested the water contact angle of GDL. The conductivity of GDL was measured by a four-probe conductivity tester (RTS-8; Guangzhou Four Probe Technology Co., Ltd., Guangzhou, China). The surface roughness of GDL was measured using a 3D morphometer (Olympus LEXT OLS4500, Tokyo, Japan). The porosity and pore size distribution were measured by a mercury injection instrument (Auto pore IV 9500V1.09; Qingdao Bolian Foucault Innovation Technology Co., Ltd., Qingdao, China). The polarization curve, power density curve, and electrochemical impedance spectrum of PEMFC (Scribner Associates, NC, 850e, Southern Pines, NC, USA) were used to test the electrochemical performance of GDL. An electronic scale (accuracy 0.01 g, Zhejiang Kaifeng Co., Ltd., Yongkang, China) was used to weigh the mass of carbon black and sodium bicarbonate. An airbrush (W-71G; Fujiwara, Osaka, Japan) was used to spray the prepared solution. The thickness of GDL was measured by the digital display thickness measuring instrument (CH-01BM, accuracy 0.001 mm Qingdao Aice Technology Co., Ltd., Qingdao, China). An electric heating plate (DB-XAB; Shanghai Lichenbang Instrument Technology Co., Ltd., Shanghai, China) removed the anhydrous ethanol while spraying the solution. An ultrasonic cleaning machine (CH-01BM; Suzhou Chuanghui Electronics Co., Ltd., Suzhou, China) was used for ultrasonic dispersion, and a magnetic stirrer (84-1A; Jinxinrui Instrument Co., Ltd., Changzhou, China) was for stirring solutions.

## 3. PEMFC Performance Test Process

### 3.1. Polarization and Power Density Tests

The current density, power density, and voltage drop losses of PEMFC under operating conditions were most accurately reflected by the polarization and power density curves. All the prepared GDL and commercial CCM were assembled into membrane electrodes in this experiment. The electrochemical test of the fuel cell was carried out in the hydrogen-air environment, H_2_ (purity 99.9%) was passed into the anode, and the air was passed into the cathode. The test conditions were 40% humidification (fuel cell temperature 80 °C, cathode and anode gas temperature 58.9 °C), 60% humidification (fuel cell temperature 80 °C, cathode and anode gas temperature 60 °C), 100% humidification (fuel cell temperature 80 °C, cathode and anode gas temperature 80 °C).

### 3.2. Electrochemical Impedance Tests

Electrochemical impedance spectroscopy (EIS) is a test technique that can accurately and lossless acquire various internal losses of fuel cells within a short period of time. In this study, the EIS was performed with a current density of J = 2 A/cm^2,^ and the fuel cell was repeatedly swept at 10% DC with a frequency of 1–10,000 Hz, with the different GDLs under the same conditions.

## 4. Results and Discussion

### 4.1. Single Cell Performances

#### 4.1.1. Polarization Test Analysis

**Figure 3:**
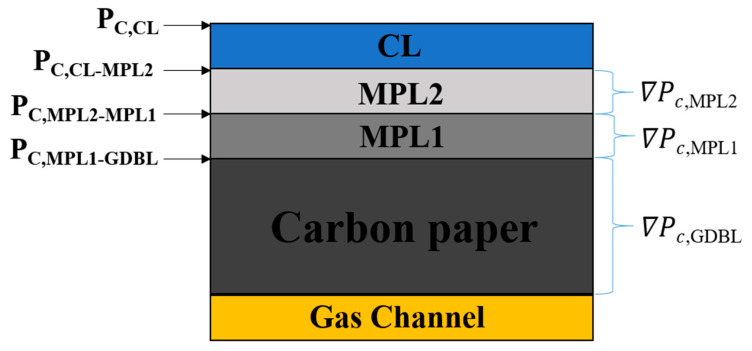
Capillary press gradient through the GDL.

Due to the low operating temperature of PEMFC, the water form produced at the cathode CL was converted to liquid water. The structural design of the GDL determines the magnitude and distribution of its internal capillary pressure, which primarily drives the discharge of liquid water inside the GDL [31,32,33]. Figure 3 shows the schematic diagram of the capillary pressure gradient through the GDL.

The capillary pressure is

(1) $$P_{C}=P_{l,H_{2}O}-P_{g}=\frac{\sigma \cos\theta_{c}}{{\left(K/\varepsilon \right)}^{1/2}}J(S)$$

(2) $$J(s)=\left\{\begin{array}{l} 1.417(1-s)-2.120{\left(1-s\right)}^{2}+1.263{\left(1-s\right)}^{3},\;\theta_{c}<90{}^{\circ}\\ 1.417-2.120s^{2}+1.263s^{3}\;\;\;\;\;\;\;\;\;\;\;\;\;\;\;\;\;\;\;\;\;\;\;\;\;\;\;\;\;\;\;\;,\;\theta_{c}<90{}^{\circ} \end{array}\right.$$

The radius of the capillary tube is

(3) $$r_{c}=2{\left(K/\varepsilon \right)}^{1/2}=\frac{-2\sigma \cos\theta_{c}}{P_{C}}$$

where *P_c_* is the capillary pressure, *P_l_* is the liquid phase pressure, *P_g_* is the gas phase pressure, σ is the surface tension, *θ_c_* is the water contact angle, *ε* is the porosity, *K* is the permeability, *J*(*s*) is the Leverett J function, *s* is the liquid water saturation, and *r_c_* is the radius of the pore.

Therefore, when the PEMFC was at a smaller operating current density, the cathode produced less liquid water, and consequently, the gas phase pressure was more significant than the liquid phase pressure, i.e., *P_c_* < 0. It is known from Equation (1) that when the water contact angle was $0{}^{\circ}\leq \theta_{c}\leq 90{}^{\circ}$, liquid water could quickly enter the hydrophilic pore. The smaller the pore size, the greater the capillary pressure, and the cathode water is more easily discharged. When the PEMFC was working at high current density, the cathode CL generated a large amount of water, and the liquid phase pressure was higher than the gas phase pressure, i.e., $P_{C}>0$. The hydrophilic pores cannot meet the drainage requirements of the GDL, and the liquid water will preferentially enter the hydrophobic pores. Since the critical *P_c_* into the larger hydrophobic pore was lower than the crucial *P_c_* into the smaller hydrophobic pore, the liquid water preferentially penetrated the larger hydrophobic pore. Therefore, liquid water at high current densities will be transported in pore diameters of more than 20 μm. At high current density, the cathode CL produced a large amount of liquid water, which at this time occupied a large number of pores, and if the reacting gas (O_2_) maintains normal gas transport between the GDL and the CL, it can only pass through the hydrophobic mesopores.

(4) $$\nabla P_{C,MPL2}=\frac{P_{C,CL-MPL2}-P_{C,MPL2-MPL1}}{\delta_{MPL2}}$$

(5) $$\nabla P_{C,MPL1}=\frac{P_{C,MPL2-MPL1}-P_{C,MPL1-GDBL}}{\delta_{MPL1}}$$

(6) $$\nabla P_{C,GDBL}=\frac{P_{C,MPL1-GDBL}-P_{C,GDBL-GasChannel}}{\delta_{GDBL}}$$

The above analysis shows that the capillary pressure, water contact angle, pore size, and porosity have an inseparable relationship. It is known from Equation (3) that the smaller the pore size, the higher the capillary pressure. From this, it can be concluded that $\nabla P_{C-MPL2}>\nabla P_{C-MPL1}$ in GDL03, $\nabla P_{C-MPL2}<\nabla P_{C-MPL1}$ in GDL04, $\nabla P_{C-MPL2}=\nabla P_{C-MPL1}$ in GDL01, and GDL02. The pore size increased from MPL2 to GDBL step by step to realize the capillary pressure difference in the thickness direction of GDL03 by optimizing the pore size structure in MPL. It was proved that the gradient pore size structure of GDL03 drove the capillary pressure gradually to increase from GDBL to CL/MPL interface. This promoted the discharge of water produced by the cathode, an important reason why the electrochemical performance of sample GDL03 was better than other samples.

Table 2 shows the pore size content of GDL for different pore size intervals. The laboratory-prepared GDL had a 7–20 μm pore size for gas transport and a 20–50 μm pore size for water management than the commercial GDL29BC. Significantly the increase of 20–50 μm pore size was more conducive to the discharge of water from the high current density of the cathode GDL to prevent flooding. Commercial GDL29BC had large pores primarily concentrated in the 40–100 μm range and had a broad distribution range. The GDL03 had more pore sizes in the field of 7–20 µm and 20–50 µm, thus redistributing the pore size range of GDL. This was another important reason why the electrochemical performance of sample GDL03 was better than other samples.

**Figure 4:**
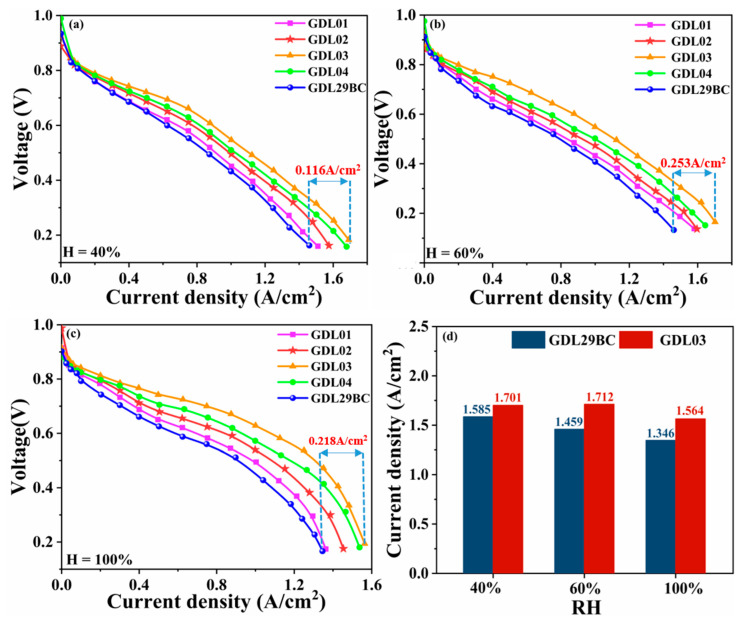
The polarization curve of commercial GDL29BC and laboratory prepared GDL. (**a**) 40% humidification; (**b**) 60% humidification; (**c**) 100% humidification; (**d**) limiting current density of GDL03 and GDL29BC at 40%, 60% and 100% humidification.

Figure 4 shows the polarization curves of commercial GDL29BC and laboratory prepared GDL at 40%, 60%, and 100% humidity. The difference in potential drops for different GDLs occurred at low current densities, which was governed by the activation region. The electrochemical activation loss was mainly due to the kinetic limitation of the electrochemical reaction on the electrode surface, and the electrochemical activation loss was directly related to the electrochemical reaction rate. The electrochemical reduction kinetics of O_2_ on the cathode side was very slow. The activation loss increased rapidly with the increase of current density in the low current density region. As seen in Figure 4a, under the condition of 40% humidity, the single-cell performance of four GDLs prepared in the laboratory was better than commercial GDL29BC. The GDL03 single cell had the best performance with a limiting current density of 1.701 A/cm^2^, while commercial GDL29BC only had a limiting current density of 1.585 A/cm^2^. GDL01, GDL02, and GDL04 also showed good single-cell performance with limiting current densities of 1.511, 1.574, and 1.676 A/cm^2^, respectively. The improved performance was contributed by the enhanced activation process on the electrode. Figure 4b displays fuel cell polarization curves of commercial GDL29BC and laboratory prepared GDL under 60% humidity. The GDL03 had the best single fuel cell performance with a limiting current density of 1.712 A/cm^2^ compared to 1.459 A/cm^2^ for commercial GDL29BC, which improved performance by 17.34%. As seen in Figure 4c, the single-cell performance of each GDL showed significant differences at 100% humidity, which were reflected not only in the performance differences between commercial GDL29BC and laboratory-prepared GDLs but also between laboratory-prepared GDLs. The performance difference became more and more pronounced as the current density increased. GDL03 and GDL04 with dual-stage microporous layers had better electrochemical properties than GDL01 and GDL02 with a single microporous layer. The two-stage MPL provided a higher capillary pressure difference to facilitate water and gas transfer. The GDL03 had the best single fuel cell performance with a maximum current density of 1.564 A/cm^2^ compared to 1.346 A/cm^2^ for commercial GDL29BC. As seen in Figure 4c, GDL01 showed little performance difference with commercial GDL29BC at 100% humidity, while GDL02, GDL03, and GDL04 showed significant performance differences with commercial GDL29BC. In sum, the results showed that the laboratory prepared GDL performed better than the commercial GDL29BC under three wetting conditions. In particular, the GDL03 sample had excellent output performance under three wetting conditions. Given that, the GDL03 sample displayed the best single-cell performance at 60% humidity with a maximum current density of 1.712 A/cm^2^. The CL layer was the main factor affecting the performance of PEMFC under three humidity conditions. The CL layer accelerated electrode reaction kinetic processes and reduced the activated polarization’s over potential. As the reaction progressed, the response entered the ohmic polarization region. Ohmic polarization is a voltage drop due to the resistance during ion and electron transport in the fuel cell, where MEA is the main component of the ohmic resistance. When the polarization curve entered the concentration polarization region (higher current density), the reaction rate was significant, and a large amount of product water was produced. If generated water was not discharged in time, gas (O_2_) did not reach the electrode surface smoothly, and the concentration of reactants near the electrode surface decreased rapidly. The analysis revealed that the 20–50 μm pore size range was more conducive to water transportation. Hence, GDL03 performed best at high current densities because (a) The gradient pore size of GDL03 provided a higher capillary pressure difference, and the capillary pressure gradually increased from the GDBL to CL/MPL interface. (b) The GDL03 had more pores in the 20–50 μm pore size range that provided water and gas transfer. The comparison of polarization curves proved that a two-stage MPL with a reasonable gradient pore size structure could be more beneficial in improving the performance of PEMFC.

#### 4.1.2. Power Density Test Analysis

**Figure 5:**
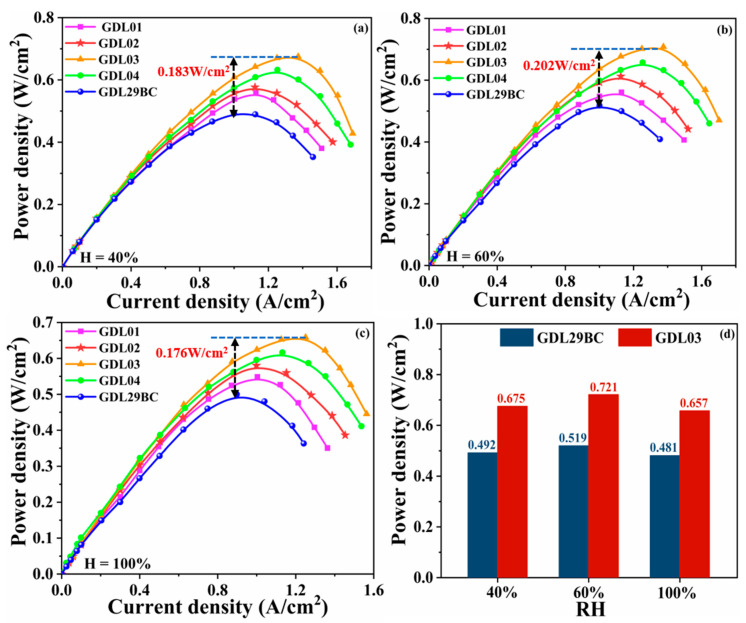
The power density of commercial GDL29BC and laboratory prepared GDL. (**a**) 40% humidification; (**b**) 60% humidification; (**c**) 100% humidification; (**d**) limiting power density of GDL03 and GDL29BC at 40%, 60% and 100% humidification.

Figure 5 shows the power density curves of commercial GDL29BC and laboratory prepared GDL obtained at 40%, 60%, and 100% humidity, where it can be seen that GDL03 exhibited the best power density performance, especially the highest power density of 0.721 W/cm^2^ at 60% humidity. There was very little performance difference between the GDL when the current density was between 0 and 0.2 A/cm^2^. The performance gap between GDLs increased as the electrochemical process advanced, particularly at 100% humidity. GDL03 and GDL04 with dual-stage microporous layers had better electrochemical properties than GDL01 and GDL02 with a single microporous layer. The two-stage MPL provided a higher capillary pressure difference to facilitate water and gas transfer. It can be seen from Figure 5d that under three humidity levels, the highest power density of the GDL03 was 0.675, 0.721, and 0.657 W/cm^2^, as opposed to the commercial GDL29BC’s 0.492, 0.519, and 0.481 W/cm^2^ maximum power densities. Figure 5a–c shows that the maximum power density of GDL03 is increased by 0.183, 0.202, and 0.176 W/cm^2^, respectively, compared to GDL29BC. The reasons for these results were as follows: (a) GDL03 had a larger 7–20 μm pore size beneficial for air and oxygen transport, and a larger 20–50 μm pore size beneficial for water management, especially at high current densities, fuel cell cathodes produced large amounts of water that occupied 20–50 μm pore size. At this time, reaction gas (O_2_) can be transported through a pore size of 7–20 μm, and reasonable distribution of pore size essentially determines the performance of a single cell. (b) Gradient pore size design changed the transmission path of water and gas. Carbon paper as GDBL had a larger pore size, and its pore size was mainly distributed in 50–80 μm, while the pore size of MPL was smaller. A transition layer with a reasonable pore size was added between the two to achieve a smooth transition from GDBL to MPL. (c) The gradient pore size structure of GDL03 driven the capillary pressure gradually increased from GDBL to CL/MPL interface. This promoted the discharge of water produced by the cathode. Eventually, the power density of a single cell increased.

### 4.2. Electrochemical Impedance Spectroscopy Test

**Figure 6:**
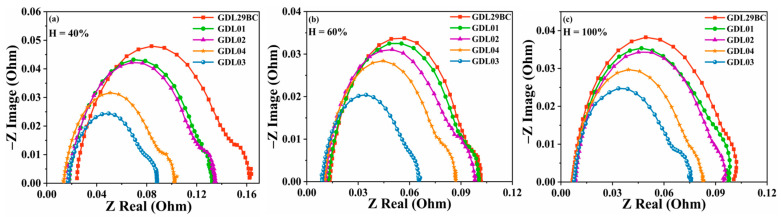
EIS of commercial GDL29BC and laboratory prepared GDL. (**a**) 40% humidification; (**b**) 60% humidification; (**c**) 100 humidification.

EIS is a dynamic measurement technique in which when a small amplitude AC sine wave of varying frequency is applied to the spot or current of an electrode, the corresponding current will change accordingly. The impedance spectrum is usually obtained by applying a sinusoidal voltage perturbation to the system at a certain current density and measuring the variation of the resulting current with a time between 1 and 10,000 Hz. The form of loss of fuel cells at different current densities was different. Activation loss occurred mainly at low current densities; ohmic losses were primarily caused by the resistance generated during the binding of fuel cell components and impedance generated by proton exchange membrane transport; material transport impedance occurred mainly at high current densities. Results of the EIS tests are displayed in Figure 6 for both laboratory prepared GDL and commercial GDL29BC. The EIS test was performed at a current density of J = 2 A/cm^2^. The laboratory prepared GDLs exhibited smaller arcs than commercial GDL29BC in three humidity conditions. The high-frequency impedance dominated the fuel cell impedance. This was because high-frequency impedance was associated with electrochemical and charge transfer processes occurring at the electrode-electrolyte interface, while low-frequency impedance was more related to mass transfer contributions and reactant transport to the electrode surface. Low-frequency arcs reflected the impedance of the activation process due to mass transfer limitations. The laboratory- prepared GDL had a graded pore-size structure with better water and gas transport at high current densities. In contrast, commercial GDL29BC was designed by a coating process with a single pore size. The material transfer performance of GDL03 was superior to the other three laboratory prepared GDLs, and GDL03 had the lowest impedance among the three humidified situations. The reason is that the pore size distribution of GDL03 is more reasonable, especially in the pore size range of 7–20 μm and 20–50 μm. The high current density favors the timely discharge of cathode water, and O_2_ can reach cathode CL and react smoothly with H_2_, which reduces the material transmission impedance at high power densities. At the same time, GDL03 has lower ohmic loss and cathodic activation loss compared to other GDLs prepared in the laboratory, as well as GDL29BC. In the high-frequency region, the processes of mass transfer and charge transfer mainly occur. The response summit caused by the electrolyte resistance and the transfer resistance is clearly manifested. As the frequency increases, the impedance amplitude shows a gradual increase. This is because the mass transfer and charge transfer occur interleaved, causing the polarization layer to form, resulting in an increase in system impedance. Thus, GDL03 has a lower electrochemical impedance and a higher output performance at three humidity levels.

### 4.3. Surface Hydrophobicity of GDLs

**Figure 7:**
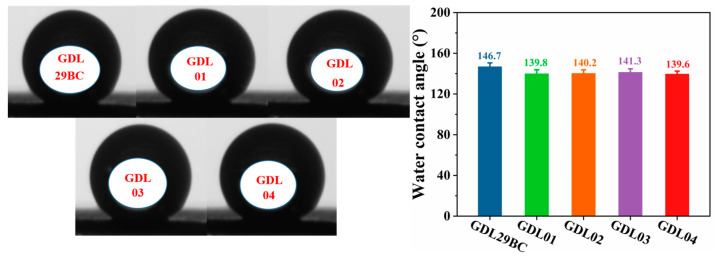
Commercial GDL29BC and laboratory-prepared gas diffusion layer water contact angle test results.

In order to maintain sufficient hydration between CL and PEM, facilitating the transport and discharge of cathode water from PEMFC at high current densities and preventing flooding, MPL should have good hydrophobicity. For this reason, MPL commonly used PTFE as a hydrophobic agent to construct internal water and air transport channels. Figure 7 shows the water contact angle test results for the commercial GDL and laboratory prepared GDL. The water contact angle of GDL29BC was 146.7° (*σ* = 3.5°), which reflected the good hydrophobic property of commercial GDL29BC. The water contact angles of GDL01, GDL02, GDL03 and GDL04 prepared in the laboratory were 139.8° (*σ* = 3.8°), 140.2° (*σ* = 3.5°), 141.3° (*σ* = 3.3°) and 139.6° (*σ* = 3.0°), respectively. The difference in water contact angles of the four laboratory prepared GDLs was slight, mainly due to the uniform PTFE content (30%) used in the preparation process. Although the hydrophobicity was marginally lower than that of commercial GDL29BC, the difference was insignificant. The reasons supporting this analysis were as follows: (a) The hydrophobicity of MPL mainly depended on the hydrophobic agent type and the hydrophobic agent’s content. The GDL prepared in this experiment contains 30% hydrophobic PTFE content, while commercial GDL29BC contains more content of hydrophobic agents. Therefore, commercial GDL29BC has better hydrophobicity. (b) The surface roughness values of the laboratory sprayed GDL range from 2.4 to 3.0 μm, while the commercial GDL29BC has a smoother surface roughness value of only 1.184 μm. The larger the roughness, the lower the surface water contact angle. This is because the surface roughness can lead to an increase in the surface of the material’s tiny pores, the formation of a larger effective surface area. Thereby increasing the surface area in contact with the liquid improves the interaction between the liquid and the material surface, so that the liquid is easy to lose penetration into the interior of the material, thereby reducing the water contact angle. 

### 4.4. Electrical Resistivity of GDLs

**Figure 8:**
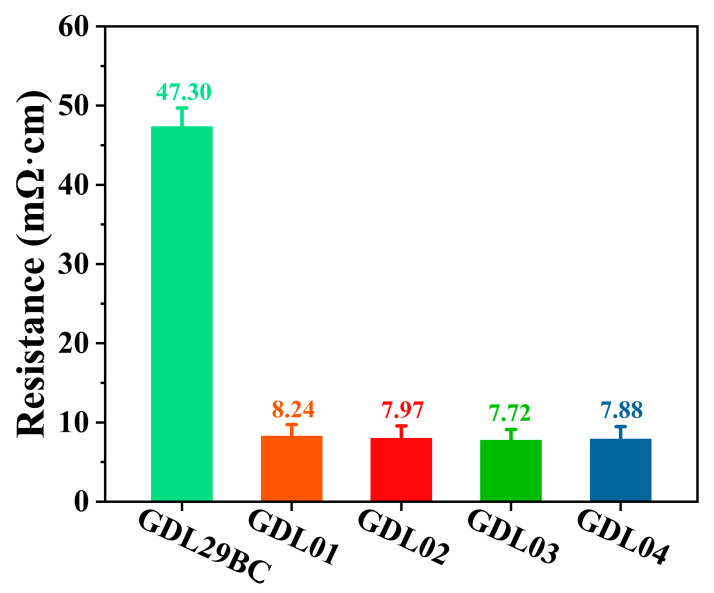
The resistivity of commercial GDL29BC and laboratory-prepared gas diffusion layer.

Resistivity was an essential factor in the performance of GDL. The higher the resistance value of GDL, the more significant the ohmic impedance of fuel cells. The lower the mass transfer efficiency, the more considerable the voltage loss at the same current density is. This ultimately led to a decrease in the power density of fuel cells and the efficiency of power generation. Figure 8 shows resistivity test results for commercial GDL29BC and GDL prepared in this study. It can be seen from the figure that the resistivity of commercial GDL29BC was 47.30 mΩ·cm (*σ* = 2.40 mΩ·cm), while the resistivity of GDL01, GDL02, GDL03, and GDL04 prepared in the laboratory was 8.24 (*σ* = 1.50), 7.97 (*σ* = 1.60), 7.72 (*σ* = 1.40), and 7.88 (*σ* = 1.60) mΩ·cm, respectively, which was much lower than the commercial GDL29BC. Consequently, a high level of electron transfer efficiency decreased ohmic impedance and enhanced fuel cell output performance. The following factors contributed to this: (a) The PTFE content of commercial GDL29BC was higher than the GDL prepared in the laboratory. Although PTFE improved the hydrophobic properties of GDL, as an insulating material, it affected the binding between carbon black and increased the resistivity of GDL. (b) The MPL of commercial GDL29BC was prepared by a coating process, while in this study, MPL prepared by the spraying process had a more considerable effective contact between conducting carbon black in the thickness direction. However, there was a slight difference between the in-plane resistances of laboratory-prepared samples. On the one hand, the reason for this discrepancy may be that the GDL surface was not consistent due to different NaHCO_3_ content; On the other hand, the effective contact area between carbon black and carbon black was also changed after the removal of NaHCO_3_, so in-plane resistances of GDL were different.

### 4.5. SEM Characterization of GDLs

**Figure 9:**
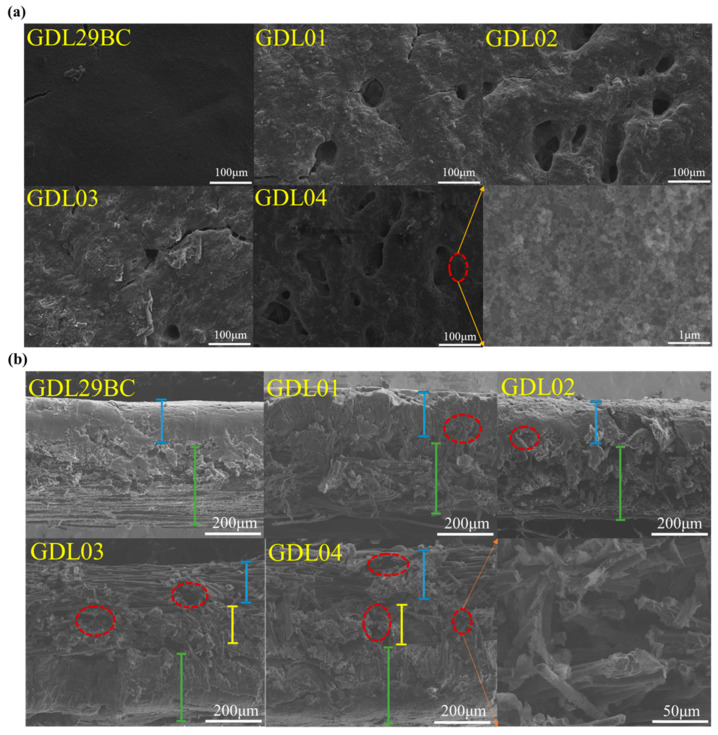
(**a**) Surface and (**b**) section morphology of commercial GDL29BC and laboratory-prepared gas diffusion layer.

Surface morphologies of commercial GDL29BC and laboratory-prepared GDL are displayed in Figure 9a. Commercial GDL29BC had a relatively apparent smooth surface compared to the surface of laboratory-prepared GDL. This was because commercial GDL29BC was prepared by the smearing method, where carbon and carbonaceous particles were uniformly attached to form an excellent planar structure. However, the GDL prepared in this study was made by spraying. The distribution of carbon black particles was not uniform. At the same time, many pores were created after NaHCO_3_ was broken down by heat, resulting in a large surface roughness. The apparent surface roughness of GDL02 was larger than GDL01 because the MPL of GDL02 contained more NaHCO_3_ particles. Thermal decomposition of NaHCO_3_ at high temperatures led to significant differences in the surface of MPL. Hence, the surfaces of GDL01 and GDL03 seemed flatter than those of GDL02 and GDL04; this was because MPL2 of GDL01 and GDL03 contained less NaHCO_3_.

The cross-section morphologies of commercial GDL29BC and laboratory prepared GDL are displayed in Figure 9b. The part marked by the red circle is the pore in the GDL cross-sectional morphology, the blue line represents MPL2 in the GDL, the yellow line represents MPL1 in the GDL, and the green line represents GDBL in the GDL. From the figure, it can be seen that the laboratory prepared GDL had a large number of pores of different sizes on the side. These pores were created after NaHCO_3_ was broken down by heat. Both commercial GDL29BC and laboratory-prepared GDL had a distinct stratified structure in cross-section. GDL29BC, GDL01, and GDL02 were secondary layered structures consisting of carbon paper and a single layer of MPL. GDL03 and GDL04 showed a distinct tertiary hierarchical structure consisting of carbon paper, MPL1, and MPL2. In this study, the successful preparation of hierarchical MPL was demonstrated. The cross-section comparison shows that laboratory-prepared GDL had significantly more pores due to the addition of the pore-making agent NaHCO_3_.

### 4.6. Surface Roughness Characterization of GDLs

**Figure 10:**
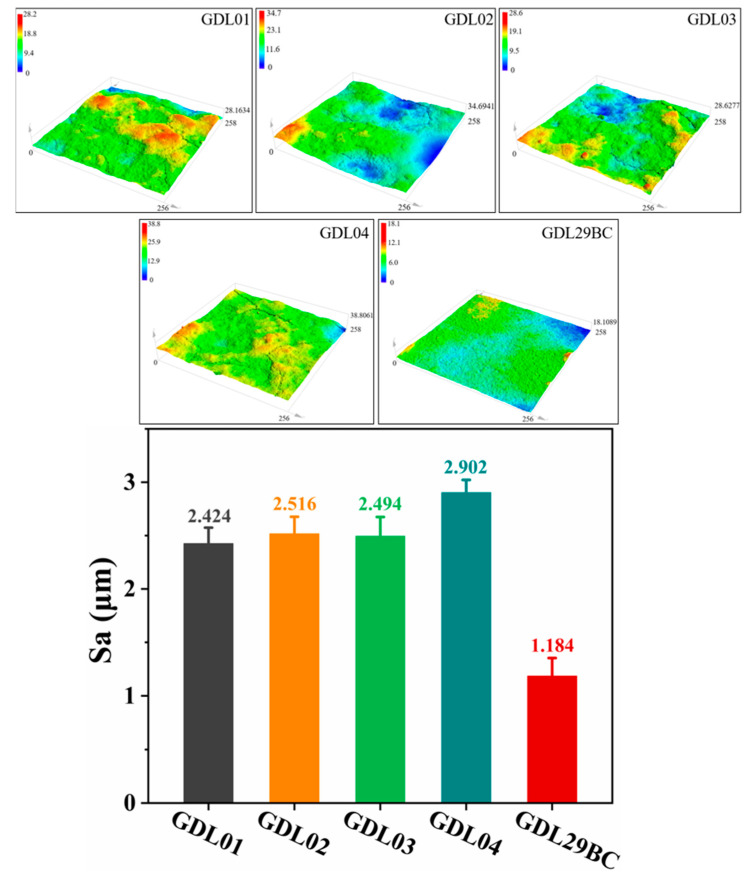
Surface roughness measurement results of commercial GDL29BC and laboratory prepared GDL.

The roughness of surface morphology affected electrical resistance between GDL and CL. The greater the surface roughness of the GDL, the greater the electrical resistance within the MEA, which involves the internal material transport capacity of PEMFC. This is because surface roughness increases the contact area between GDL and CL. From a microscopic point of view, surface roughness leads to some small-scale geometric unevenness and defects that introduce additional scattering and hindrance during current transport, making the resistivity increase. In addition, the surface roughness may change the charge distribution at the interface, the diffusion rate of cathodic reactants, and other processes that affect the electrical properties of the MEA. Figure 10 shows the surface roughness results for commercial GDL29BC and laboratory prepared GDL. The surface roughness of GDL was measured using a 3D morphometer (Olympus LEXT OLS4500, Tokyo, Japan). As can be seen from the figure, the surface of commercial GDL29BC was the flattest, with a surface roughness of only 1.184 μm (*σ* = 0.170 μm), while the surface roughness of laboratory prepared GDL was more significant. This was mainly because commercial GDL29BC was prepared by a coating process with a relatively flat surface. The laboratory made GDL was prepared by a spraying process in which carbon black particles were inhomogeneously dispersed on the surface. The surface roughness of GDL01, GDL02, GDL03, and GDL04 were 2.424 (*σ* = 0.152), 2.516 (*σ* = 0.166), 2.494 (*σ* = 0.185), 2.902 μm (*σ* = 0.122 μm), respectively. The four laboratory-prepared samples had few differences. However, the surface roughness of GDL02 and GDL04 was higher than GDL01 and GDL03. The reason was that MPL2 of GDL02 and GDL04 contained more NaHCO_3_, which left more pores after the decomposition of NaHCO_3_, resulting in greater surface roughness.

### 4.7. Porosity Characterization of GDLs

**Figure 11:**
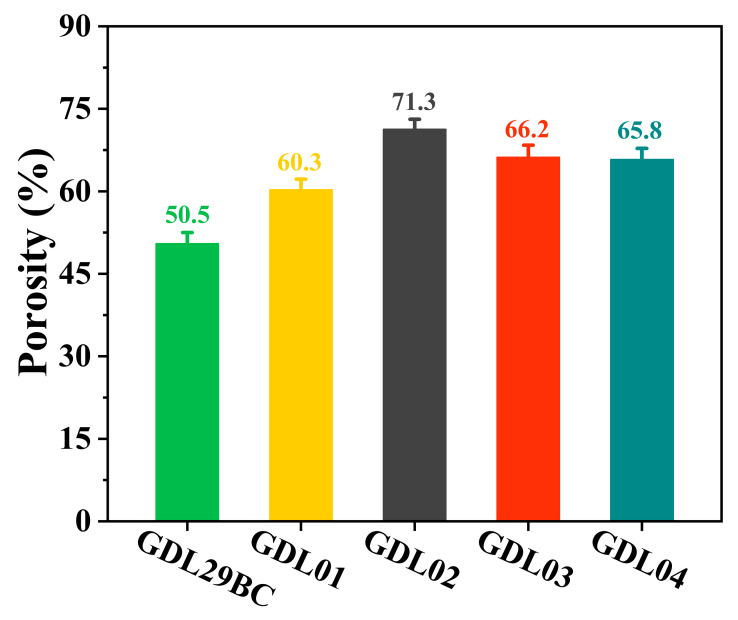
The porosity of commercial GDL29BC and laboratory prepared GDL.

The porosity affects the performance of PEMFC [34,35,36]. Since carbon paper and MPL cannot be independently tested for porosity, mercury injection was used in this study to test the porosity of commercial GDL29BC and laboratory prepared GDL. Since the base layer of commercial GDL29BC and laboratory prepared GDL was commercial carbon paper, the difference in porosity was mainly in MPL. Figure 11 shows the porosity test results for commercial GDL29BC and laboratory prepared GDL. The porosity of laboratory prepared GDL01, GDL02, GDL03, and GDL04 were 60.3% (*σ* = 1.9%), 71.3% (*σ* = 1.8%), 66.2% (*σ* = 2.2%), 65.8% (*σ* = 2.1%), respectively, but commercial GDL29BC had a porosity of 50.5% (*σ* = 2.0%). The primary causes for these results were: (a) The commercial GDL29BC’s relatively high PTFE content can block carbon paper and MPL pores and reduce porosity. (b) GDL prepared in the laboratory contained different proportions of NaHCO_3_. NaHCO_3_ was broken down and formed some pores after sintering at high temperatures, resulting in increased porosity. Therefore, the different amounts of NaHCO_3_ in the MPL were the leading cause of the porosity differences between GDL01, GDL02, GDL03, and GDL04. The porosity of GDL02 was higher than other samples because GDL02 contained more NaHCO_3_.

**Figure 12:**
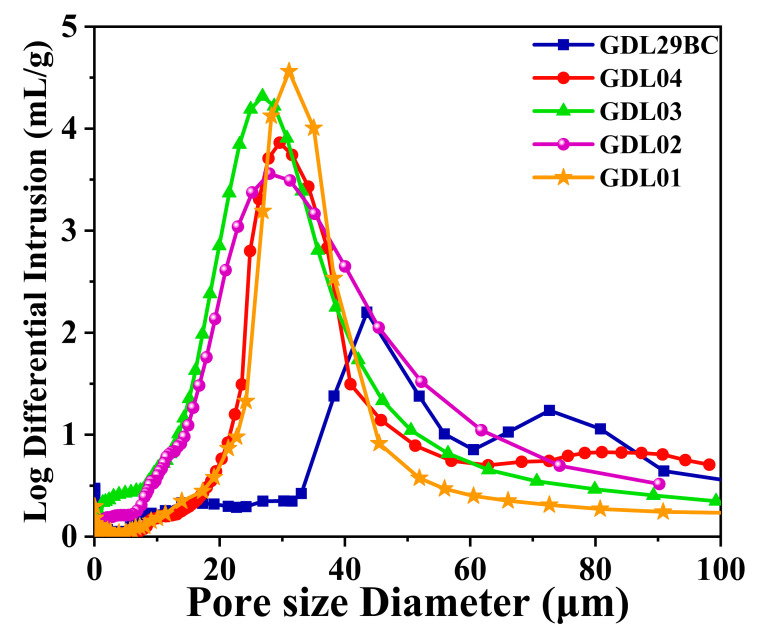
The pore-size distribution of commercial GDL29BC and laboratory-prepared GDL.

Pore size distribution played an important role in the water and gas management of PEMFC [37]. The base layer of carbon paper contained large pores but lacked the mesopores for transporting liquid water and gas. The free travel of air is about 70 nm, molecular diffusion mainly occurs in pores larger than 7 μm, and Knudsen diffusion mainly occurs in pores smaller than 7 μm. In addition, considering that the diffusion coefficient of molecular diffusion is 3 orders of magnitude higher than that of Knudsen diffusion, it is finally concluded that the pores larger than 7 μm in the GDL are more favorable for O_2_ transfer [38,39]. Figure 12 shows the pore size distribution of commercial GDL29BC and laboratory prepared GDL. The pore size distribution of GDL02 showed a prominent peak in the pore size range of 20–50 μm, while the rise of GDL01 was mainly concentrated in the pore size range of 30–40 μm, and the peak was higher. The difference between GDL03 and GDL04 was mainly reflected in that GDL03 had higher porosity in the 20–40 μm pore size range, while GDL04 had more pores in the 60–100 μm pore size range. The reason was that MPL2 of GDL03 had less NaHCO_3_, resulting in tight adhesion between carbon black particles and tiny pores. Excessive NaHCO_3_ in GDL04 caused large pores after thermal decomposition, and the number of mesoporous in pore size of 20–40 μm decreased sharply. GDL03 had a higher 7–20 μm pore size than GDL01. Figure 12 shows that the pore size of commercial GDL29BC was mainly distributed in the pore size range of 40–80 μm. The 40–80 μm pore size was less favorable for gas transfer. This was the main reason why the performance of commercial GDL29BC was lower than laboratory prepared GDL.

## 5. Conclusions

This paper reports the preparation of a two-stage MPL with a gradient pore size structure using the hydrophobic agent PTFE, the pore-making agent NaHCO_3_, and a conductive carbon black material. The physical and electrochemical properties of the gradient-structured GDL were compared with the commercial GDL29BC. It was found that the laboratory prepared GDL with gradient pore size structure not only had good hydrophobicity and conductivity but also had a more reasonable pore size distribution and stable electrochemical properties. Moreover, it has been shown that changing the porosity and pore size distribution of MPL by adding different ratios of pore-making agent ultimately increases the capillary pressure difference within the GDL. In addition to increasing the number of 7–20 μm pore sizes for air transmission, 20–50 μm pore sizes also were added for better water management. The results showed that the electrochemical performance of laboratory-prepared GDL was better than commercial GDL29BC, with a higher limiting current density and lower electrochemical impedance in a hydrogen-air environment. The maximum power density of GDL03 reached 0.675, 0.721, and 0.657 W/cm^2^ at 40%, 60%, and 100% humidity, respectively. The maximum power density of the GDL03 was increased by 37.1% at 40% humidity, 38.9% at 60% humidity, and 36.5% at 100% humidity when compared to the commercial GDL29BC. Therefore, we were able to show that the gradient structure of GDL significantly improved the electrochemical performance of PEMFC.

## Figures and Tables

**Table 1:** Parameters of different GDL samples prepared.

Samples	NaHCO_3_:Vulcan XC-72 + PTFE		MPL Thickness (μm)	PTFE (wt.%)
	MPL1	MPL2		
GDL01	1:4	1:4	60.0 ± 1.8	30.0
GDL02	1:2	1:2	60.0 ± 2.1	30.0
GDL03	1:2	1:4	60.0 ± 2.0	30.0
GDL04	1:4	1:2	60.0 ± 2.2	30.0

**Table 2:** Pore size distribution intervals of commercial GDL29BC and laboratory prepared GDL.

ML/g	7–20 μm	20–50 μm	50–100 μm
GDL29BC	2.61	6.24	9.56
GDL01	3.08	23.65	3.77
GDL02	22.90	25.40	4.22
GDL03	24.99	36.40	4.57
GDL04	8.68	31.60	7.35

## Data Availability

The data that support the findings of this study are available from the corresponding author upon reasonable request.
